# Development of QuEChERS Method for the Determination of Polycyclic Aromatic Hydrocarbons in Smoked Meat Products Using GC-MS from Qatar

**DOI:** 10.1155/2018/9206237

**Published:** 2018-07-18

**Authors:** Hussain Al-Thaiban, Nada Al-Tamimi, Murad Helaleh

**Affiliations:** Anti Doping Lab-Qatar, Toxicology Multipurpose laboratory, P.O. Box 27775, Doha, Qatar

## Abstract

A simple and fast method for the determination of PAHs in smoked meat samples was described. The QuEChERS (Z-Sep) procedure was used for sample preparation. Gas chromatograph-mass spectrometer with electron ionization (EI) was used to separate and detect the PAHs. All 16 common PAHs were analyzed successfully. Matrix-matched calibration was applied. Spiked samples were performed at 1 ng/g (*n*=10) and 10 ng/g (*n*=10) for two days. Overall recoveries of PAHs were within 74 to 117%, with RSDs within 1.15 to 37.57% and 1 and 10 ng/g wet weight for first and second day, respectively. In most of the analyzed smoked meat samples, there were no exceeded levels compared to the maximum levels declared by Commission Regulation (EU) number 835/2011. The method can be recommended for routine analysis for laboratories having a large number of samples.

## 1. Introduction

PAHs represents a group of organic compounds (hydrophobic) consisting of two or more fused aromatic rings that are ubiquitous pollutants and those can be generated during food processing [1]. PAHs containing five or more rings are considered having potential carcinogenic and genotoxic to humans and regarded as concerned to public health [2–4]. Therefore, exposure to PAHs is recommended to be as low as possible [5]. Diet is considered to be one of the highest exposures to PAHs to the most population in different countries and counts to be more than 90% [2, 6, 7]. However, high level of PAHs is not usually detected in raw foods [7].

PAHs are widely distributed in the environment and remains for a long time [8]. Benzo[a]pyrene (B[a]P) is carcinogenic and mutagenic [9] and may cause a substantial contributed to burden cancer in human [10]. However, according to scientific committee on food (SCF) [11], B[a]P was considered as a marker for the occurrence and impact of PAHs carcinogenic in food.

PAH compounds those containing five or more aromatic rings are known as “heavy molecular weight (HMW)” PAHs and those with less than five rings are known as “light molecular weight” (LMW) PAHs. Heavy and light PAHs are nonpolar compounds, showing high lipophilic nature. However, HMW PAHs is more toxic than LMW PAHs [12]. Other groups of PAHs which are not defined as carcinogens may act as synergists. As a general concept, PAHs are present in a mixture and are regarded the priority organic pollutants [12]. The fact that PAHs are present in food insists their control in food is necessary [13].

A method for determining the PAHs levels in commercial smoked meat and fish products from polish market was reported [14], PAHs were determined in grilled meat [15], in smoked fish [16] and B[a]P in smoked meat products [17, 18],in meat products smoked with different types of wood and smoking spices [19], and in Swedish smoked meat and fish [20]. PAHs in smoked ham produced at household in Serbia were reported [21]. A study was reported for the evaluation of the health risk of long-term exposure to PAHs in daily intake of smoked meat in Southwest China [22].

There are different routes of contamination on food, which include and not limited to (i) packing materials and thermal processing of food from animal origins [23] and even during home preparation and (ii) direct deposition of PAHs from weathering of fruits and vegetables [24].

The principle of sample preparation is to remove the complexities of the matrix from the sample and to make it suitable for qualitative and quantitative analysis [25]. Extraction techniques such as Soxhlet can be simple , which consumes large solvent volumes and takes long extraction times. However, the cleanup techniques to remove residues such as lipids are challenging. Standard methods for cleanup such as GPC [26], florisil [27], and silica gel [28], which required an extensive quantity of reagents, solvents, and materials, are available in the literature. A challenge for rapid extraction and clean-up method has become essential, in order to minimize the sample preparation costs and minimize consumable costs. Quenchers method was first developed by Anastassiades et al., to provide a rapid, inexpensive, and fast method for analyzing pesticides residues in fruits and vegetables [29]. The method was extended to extract pesticide and cleanup the extract from fatty foods (e.g., avocados, milk, and eggs) [30]. Quenchers method has been shown to efficiently analyze fish fillets [31–33] and shrimp [34]. The nature of Quenchers sample preparation is promising and permits its use for extraction and cleanup, and it is efficiently applicable for the analysis of wide matrices.

Quantitative analysis of PAHs from a complex matrices such as smoked meat faces three major challenges: (i) the occurrence of most PAHs at trace levels (i.e., ppb levels), (ii) the coextract of many other components from the matrices with PAHs, which leads to difficult identification in chromatographic spectrum, and (iii) the structural similarity and many of PAHs occur as isomers, which again leads to identification problem in individual PAHs [35].

Quenchers techniques replaced the complications of common, standard extraction techniques, and they provide a simple protocol for sample handling [36]. Acetonitrile was used as an extraction solvent in QuEChERS instead of acetone, which is thought to be an excellent separator from water, after the addition of salts [37, 38]. Moreover, the cleanup protocols are adopted to effectively remove the matrix-matched materials such as polar pigment, fatty acids, organic compounds, and sugar [29].

There was a growing interest in QuEChERS among researchers all over the world; the need for further investigation is mandatory for researchers in order to facilitate and develop rapid, efficient, and effective methods for different complicated matrices.

Quenchers method is basically based on extraction with acetonitrile partitioned from an aqueous matrix using MgSO_4_ and NaCl followed by cleanup using (d-SPE) with MgSO_4_ and analysis by GC-MS. Labelled d-PAHs can be used as an internal standard to compensate the analyte loss and matrix effect on chromatographic response.

One of the main obstacles in the determination of fatty food is the high-fat content (e.g., lipids, triglycerides, and fatty acids). However, the removal of lipids is important to maintain the GC system and also to allow the low detection limits (LOD). The sensitivity of the method was confirmed by the ability to detect low PAHs concentrations at the allowable permitted levels. Lipids may have severe effects, such as reproducibility, robustness, and recovery, on analyzing PAHs by GC-MS[39]. Therefore, it is essential to remove lipids prior to analyses.

The aim of the present study is to optimize and validate the applicability of Quenchers for the extraction and cleanup and finally analyzing 16 PAHs in smoked meat samples at low LOQ levels. A few studies in the literature were reported for developing an excellent analytical method utilizing QuEChERS for determining PAHs concentrations in smoked meat samples. However, both levels and distribution patterns in smoked meat product are of interest. The method was developed to cover the analysis of 2–6 rings PAHs. Precision and accuracy of the method were validated.

## 2. Materials and Methods

### 2.1. Chemicals

All 16 PAH standards, naphthalene (NAP), acenaphthylene (ACNY), acenaphthene (ACN), fluorene (FL), phenanthrene (PHEN), anthracene (ANTH), fluoranthene (FLU), pyrene (PYR), benz[a]anthracene (B[a]A), chrysene (CHRY), benzo[b]fluoranthene (B[b]F), benzo[k]fluoranthene (B[k]F), benzo[a]pyrene (B[a]P), indeno[1,2,3-cd]pyrene (I-123-cd_P), dibenz[a,h]anthracene (D[ah]A), and benzo[g,h,i]perylene (B[ghi]P), were purchased from Cambridge isotope laboratories (Frontage Road, Andover, MA, USA). The internal standard mixture: naphthalene_d8 (NAPH_d8), acenaphthylene_d8 (ACNY_d8), phenanthrene_d10 (PHEN_d10), fluoranthene_d10 (FLU_d10), pyrene_d10 (PYR_d10), benzo[a]pyrene_d12 (B[a]P_d12), and benzo[g,h,i]perylene_d12 (B[ghi]P_d12) were obtained from Cambridge isotope laboratories (Frontage Road, Andover, MA, USA).

Hexane was obtained from Sigma-Aldrich and dichloromethane (DCM) from fluka. 50 ml polypropylene conical tube (Falcon) was used for the extraction. Acetonitrile (99.9%) was obtained from Merck. Sodium chloride (NaCl) and Magnesium Sulfate (MgSO_4_) were obtained from Sigma-Aldrich. Fixed speed vortex mixer (Fisher Scientific, USA) was used for the shaking of the tubes. The centrifuge was from Thermo Scientific-SL 16 R. The QuEChERS kits used for the extraction and cleanup of meat samples were purchased from Supel TM QuE Z-Sep (12 ml tubes) (55403-U). Heating block was from Thermo Scientific-Reacti-Therm III no. TS-18824 and Turbo Vap® LV from Biotage.

### 2.2. Meat Samples

All smoked meat samples (*n*=30) were purchased from Qatar local market (Qatar, Doha). All samples were weighed before being grinded, followed by dividing them into small portions by blender to homogenize them in order to (i) increase the surface area, (ii) break down the cell structure, and (iii) ensure analytical test portion representing whole samples. However, the meat samples have to be kept cold to avoid analyte break down. Finally, ground meat was stored by placing them in aluminum foil and storing at −20°C.

### 2.3. PAHs Standard and PAH-d8

A standard mixture containing 16 PAHs solution (2000 *μ*g/ml). Two working solutions were prepared (1000 ng/ml and 50 ng/ml) with DCM, capped using crimper cap and stored in the refrigerator until it is used. Deuterated PAHs concentration of 1000 ng/ml was prepared with DCM from the original PAHs surrogate cocktail (2000 *μ*g/ml), and the vials were capped using crimper cap and stored in the moisture cabinet at room temperature until it is used.

### 2.4. Matrix-Matched Calibration

The matrix-matched calibration was used to prepare the calibration standards. It is stored in a refrigerator at 4°C. All the standards used to prepare the matrix-spiked calibration should be taken out from the refrigerator and allowed to come at room temperature prior to use, sonicated as per the manufacturer's instructions. The matrix-matched calibration was prepared by spiking the meat sample wet weight (2.0 g) with standard PAHs to obtain seven calibration points (0.5, 1.0, 5.0, 10.0, 20.0, 25.0, and 50.0 ng/g) and with the d-PAHs of 20 ng/g.

### 2.5. Preparation of PAHs QC Samples

The QC samples must be prepared from a spiking solution with the analytes of interest. The spiking should be made using standards prepared separately from those used for calibration. The QC samples were handled exactly in the same manner as the actual samples. The QC samples were analyzed by applying the same criteria for the method being evaluated. The two QC levels were at 1.0 ng/g and 10 ng/g, with 10 replicates for each concentration level.

### 2.6. Extraction and Purification of Smoked Meat Samples

**Figure 1:**
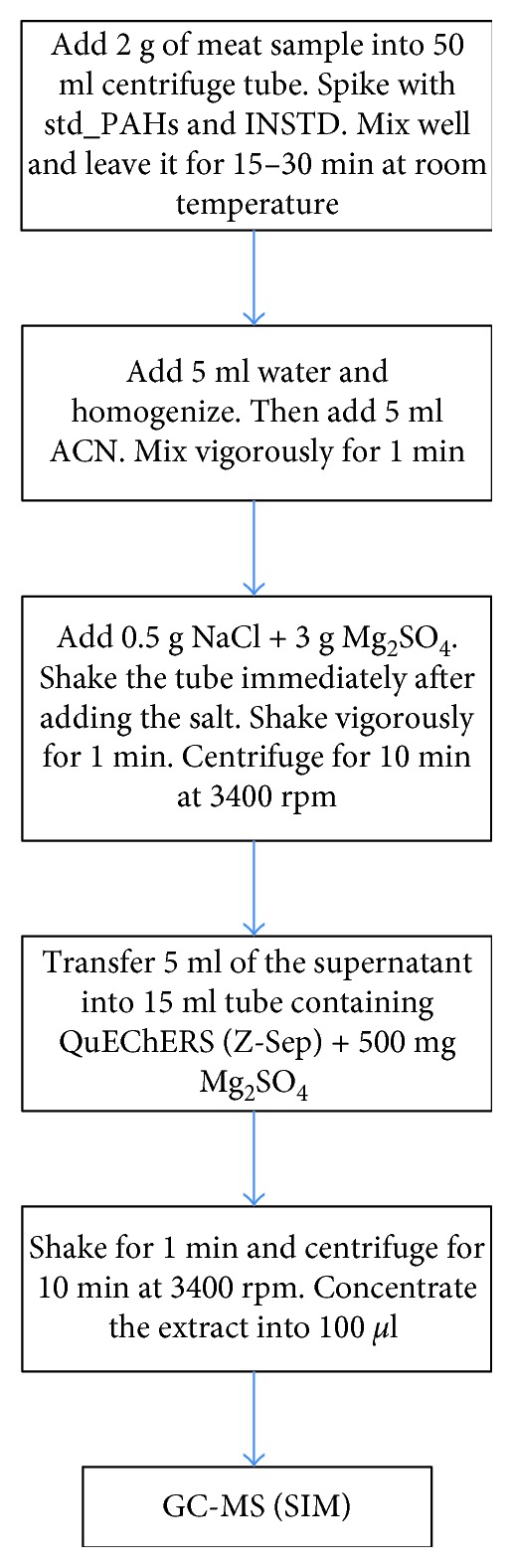
Schematic diagram for the extraction and cleanup of meat using QuEhERS method.

Chopped and stored smoked meat was taken out from the freezer, thawed at 4°C before extraction, and purified by QuEChERS method. The QuEChERS purification extract offer a fast, efficient, and accurate method for the determination of PAHs in meat samples. Two grams of smoked meat sample was added into 50 centrifuge tube and spiked with d-PAHs, mixed well, and left for 30 min at room temperature. Water was added (5 ml) and homogenized, 5 ml of ACN was added to the tube, and mixed vigorously for 1 min. Sodium chloride (0.5 g) and magnesium sulfate (3.0 g) were added to the tube; the tube was shaken immediately for 1 min after adding the salts. The content was centrifuged for 10 min at 3400 rpm (Temperature = 20°C). The supernatant was transferred into a 15 ml tube containing Quenchers (Z-Sep) + 500 mg MgSO_4_ and shaken for 1 min and centrifuged for 10 min at 3400 rpm (Temperature = 20°C). Finally, the extract was transferred into appropriate tubes and dried further using the heating block (45°C) until the volume reaches approximately 100 *µ*l. The schematic diagram of the extraction process is shown in Figure 1. Background reduction was evaluated by analysis of the extract cleaned by Z-Sep, and it shows the lowest background. The large peak eluting 7.5 to 7.9 and 19.8 to 20.5 minutes was identified or unidentified, and it did not interfere with the ions used for quantitation of PAHs.

### 2.7. GC-MS Analysis

The extract was analyzed on Agilent 6890N gas chromatography interfaced to Agilent 5975B mass spectrometer with electron ionization. The gas chromatography was connected with a 30 m HP-5MS capillary column with 0.25 mm × 0.25 *μ*m film. Helium was used as the carrier gas. The column was maintained at a constant flow rate of 1.0 ml/min, and 1 *µ*l was injected into a splitless mode and purge flow to split vent was 50 ml/min at 0.75 min.

Ion source and quadrupole temperature were set at 280°C and 180°C, respectively. Injector and transfer line were maintained at 280°C and 310°C, respectively. The column temperature was initially kept at 35°C for 1 min, ramped to 200°C at a rate of 25°C/min, ramped to 310°C at a rate of 8°C/min, and kept for 3.5 min. Selected-ion monitoring (SIM) acquisition was carried out to analyze the PAHs (*m/z*) and internal standard (*m/z*), and comparison of the base peak of each targeted PAH and d-PAH are shown in Table 1. The target ions are the quantifier ions (*m/z*) selected for each PAHs compound and are listed in Table 1. The qualifier ions were used for confirmation purpose. The peak was identified if the retention time varied within ±0.05 min compared with the calibration standard and quantified if the S/N was ≥3, and the ratio of the quantifier ion to qualifier ion was within ±20%. The sample that shows low response or less than the quantification limit was considered to be nondetected.

### 2.8. Method Linearity

The internal standard method was applied with seven matrix-matched calibrations ranging from 0.5 to 50 ng/g. The method linearity correlation coefficient (*r* ^2^) was higher than 0.995. The retention times (*R*_t_), regression coefficient (*r* ^2^), slope, and intercept of the calibration curves are summarized in Table 2. The PAH recoveries were generally higher than 70% for all PAHs. Both recoveries and precisions are acceptable and within the range for all PAH compounds. However, the proposed method meets the EU criteria that set a maximum LOD and LOQ which is equal to 0.3 *μ*g/kg and 0.9 *μ*g/kg for benzo[a]pyrene in food [40, 41]. Detection and quantification limits (LODSs and LOQs) were calculated based on the analysis of the blank response. LODS was defined as the lowest concentration of the analyte in the sample that can be measured and present at a concentration above that in the blank samples (LODS = 3 ∗ SD). LOQ was defined as the lowest concentration of the analyte that can be determined at an acceptable level of uncertainty, and usually, it was the lowest point in the calibration graph (European guideline). Analytes are considered to be quantitative when ion signal-to-noise (S/N) ≥ 3 with ±20%. All samples met the criteria but having S/N < 3 were considered to be <LOQ, while those failed the criteria were considered as not detected (nd). Method detection limits (LODSs) and limit of quantitation (LOQs) are listed in Table 2.

### 2.9. Recovery and RSDs

The recovery of (*n*=10) replicates at two levels (1 ng/g and 10 ng/g) is calculated and reported in Table 3. The recovery test was repeated on two different days. The calculated recovery and RSDs (%) are summarized in Table 3. The result shows very good recovery and excellent RSDs (%). The recovery for all sets of 10 replicates was in the average range of 74–117%. The lower spiking level was selected in the test in order to define and to check the capability of the method to detect the PAHs in meat sample at 1 ng/g. The spiking levels covered the entire range, and the recovery did not differ at the lowest and the highest concentrations. Three types of smoked meat were used to perform the recovery test, including beef roasted smoked [1]; smoked turkey breast [29], and Al-Tag beef M smoked [42]. However, there was no influence on the PAHs determination when using different types of smoked meat.

However, according to Commission Regulation (EC) number 1881/2006 and (EC) 333/2007 [40, 41, 43], the LOD for PAHs in meat is 0.3 ng/g and the LOQ was 0.9 ng/g wet weight and the recovery range of the method should range from 50 to 120%, which clearly indicates that the method was validated according to the regulation and complies with the recommendation criteria. The validated QuEChERS method selected for PAHs gives excellent recoveries, repeatability, reproducibility, and sensitivity.

## 3. Results and Discussion

In order to obtain the best recovery of the QuEChERS method. All related parameters were optimized. Results of the optimized parameters are discussed. Quenchers is a method of choice for processing samples because it is quick, easy, and inexpensive. The first step was by hydrating the meat samples, and then extracting with acetonitrile followed by partitioning with salts. Hydration steps are important in QuEChERS extraction in order to make partitioning effective [37, 44]. Acetonitrile is an effective solvent and can result in low coextracted substances for some matrices [44, 45].

Validation of the method was performed, by checking the spike recoveries for meat samples, and determined in 10 replicates for each of two levels for two different days. Method detection limits and limits of quantitation for all 16 PAHs were determined using 10 blank replicates with calculating the standard deviations (SDs).

**Figure 2:**
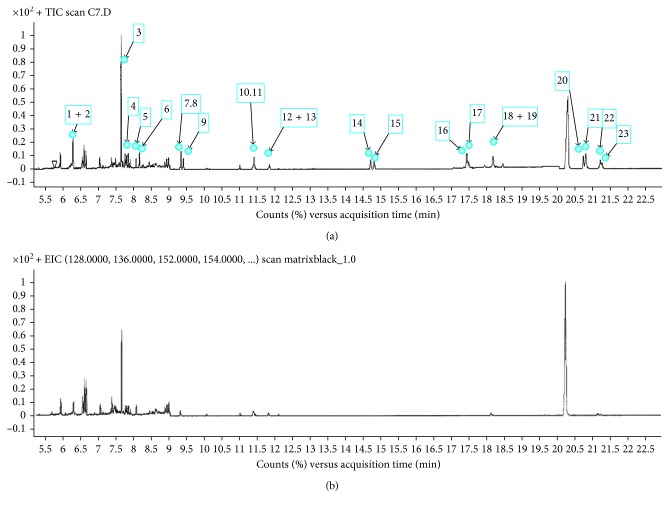
The selected-ion monitoring (SIM) obtained by Z-Sep QUE for meat samples under the selected parameters: (a) spike with 50 ng/g of 16 PAHs and I.S. (1 + 2) Naphthalene + Naph_d8, (3) acenaphylene_d8, (4) acenaphthylene, (5) acenaphthene, (6) fluorine (7 + 8) phenanthrene + Phen_d10, (9) anthracene, (10 + 11) fluoranthene + Fluro_d10, (12 + 13) pyrene + Pyr_d10, (14) benzo[a]anthracene, (15) chrysene, (16) benzo[b]fluoranthenem, (17) benzo[k]fluoranthene, (18+19) benzo[a]pyrene + B[a]P_d12, (20) indeno[1,2,3-cd]pyrene, (21) dibenz[a,h]anthracene, (22) benzo[g,h,i]perylene_d12, and (23) benzo[g,h,i]perylene. (b) Nonspike meat sample.

The GC-MS in SIM mode was shown for monitoring PAHs at a very low concentration in meat samples.  Figure 2 shows the chromatogram obtained in GC-MS (SIM) mode for meat samples (Figure 2(a)) when fortified with 16 PAHs at 50 ng/g level and for nonfortified sample (Figure 2(b)). The chromatogram separation was clean, excellent, and can be achieved without the effect of the sample matrix interferences.

### 3.1. Effect of Salts in the Extraction

Magnesium sulfate (MgSO_4_) was used as a drying agent to ensure a phase separation between organic solvent and water. Z-Sep QUE reduces concentration of fat, proteins, and other matrix components. Combination of Z-Sep and MgSO_4_ effectively removes polar matrix and water. Acetonitrile liquid-liquid partitioning is done by adding MgSO_4_ and NaCl, however, MgSO_4_ and NaCl generate sample extraction temperature of 45–50°C that persisted for the duration of the extraction. NaCl control the solvent to be removed in contact with the sample, making it to be more effective in the dissolution of analytes and facilitate the partitioning of the analytes from aqueous to the organic layer. The nonpolar PAHs with hydrophobic interaction, with pi-bond being involved, when extracted with relatively polar solvent (i.e., ACN) pi-bond and linear in geometry gave slightly better extraction. The geometry of the solvent should allow maximum interaction with the analyte besides its polarity.

Increase in salt allows greater phase separation. However, amount of salts used can also have effectiveness on the extraction system. Therefore, the role of the salt is to regulate the polarity of the matrix. Anastassiades et al. [29] showed that the use of MgSO_4_ to remove the excess of water and to provide an exothermic reaction improves the extraction process.

Furthermore, the addition of salt increases the temperature, lowering the activation energy, decreasing the viscosity of the solvent, and finally increasing the interaction of the solvent matrix [46]. Different amounts of salt were used and the optimal amount of 3.0 g of MgSO_4_ and 0.5 g of NaCl were selected throughout the experiment which gives the highest intensity response for the extracted PAHs from meat samples.

### 3.2. Effect of Solvent

As advisable, the solvent must be less expensive, compatible with analytical instrument and environment-friendly [29]. However, acetonitrile (ACN) and ethyl acetate have been largely used to extract polar to nonpolar compounds [42]. The solvent volume can play an essential role in recovery and must be in sufficient quantity to allow the full immersion of the sample into maximum solvent-analyte interaction. Different amounts of ACN were tested: 2.5, 5.0, 7.5, 10, and 15 ml. It was found that the highest peak intensity and the maximum recovery were obtained at 5 ml ACN. ACN provides a cleaner chromatogram and is considered to be one of the most selective solvents, and it has advantage over most other solvents used in QuEChERS technique [29]. However, ACN provide easier separators from water compared with other solvents used in QuEChERS in the presence of salts, providing a good phase separation which prevents interaction of polar matrix [36].

### 3.3. Effect of Centrifuge Time and Speed

The results obtained shows that excellent recovery of PAHs at 10 min, which was chosen as the optimal time for centrifuge. A centrifuge of 3400 rpm was found to be sufficient to obtain a good recovery of PAHs. The centrifuge facilitates the solvent to be more in contact with meat sample, provides more effectiveness in dissolution of the analyte [47], and hence reduces the time required for extraction.

### 3.4. Effect of Water

For ACN salting out or partitioning to occur, we must have percentage of water associated with the sample. Addition of water creating aqueous environment within the sample reduces the potential for lipids to impact extraction efficiency and minimize the fat extract. Different amounts of water were tested (2.5, 5.0, 7.5, 10, and 15 ml). It was found that the highest peak intensity and the maximum recovery were obtained with 5 ml and 7.5 ml of water, and by increasing the volume, peak intensity starts decreasing.

### 3.5. Types of Sorbent Used

Three types of cleaning sorbents were used in the extraction of meat samples. The sorbent has different effect on recovery and selectivity. The effect of sorbent types was evaluated based on the recovery achieved. Z-Sep/C18 (combination of Z-Sep and Discovery® DSC-18 particles) was used, and the recovery varied from 14 to 104%; lower recovery was observed for the PAHs having 5 and 6 rings. Another sorbent that is Z-Sep+ (dual bonded C18 and zirconium silica) was tested, and the recovery ranged from 46 to 126%, and the lower recovery was observed for the PAHs having 6 rings. The lower recoveries of PAHs having 5 and 6 rings could be due to limited solubility of these compounds in acetonitrile. However, the Z-Sep is a zirconium-coated silica (recommended for highly hydrophobic analytes such as PAHs and PCBs) yielded the highest average recoveries, and a few matrix interferences were observed in the first few minutes of the GC-MS run. Therefore, Z-Sep QUE was used throughout the experiment.

### 3.6. Analysis of Real Samples

Seven out of 16 PAHs have been categorized as probable human carcinogens. These are benzo[a]anthracene, benzo[b]fluoranthene, benzo[k]fluoranthene, chrysene, benzo[a]pyrene, dibenzo[a,h]anthracene, and indeno[1,2,3-cd]pyrene [26, 48]. The European Union has setup maximum levels of 2 ng/g wet weight for benzo[a]pyrene and considered to be a marker for carcinogenic risk [49]. However, the European Food Safety Authority [50] declared that benzo[a]pyrene is not a suitable marker for the occurrence and toxicity of PAHs in food. In 2012, four groups of PAHs were considered to provide many values to carcinogenicity, and they are benzo[a]anthracene, benzo[b]fluoranthene, chrysene, and benzo[a]pyrene [51].

In order to verify the effectiveness of the method, different types of smoked meat samples (*n*=30) were analyzed using the optimal parameters. The results of the smoked meat samples are presented in Table 4.

Noncarcinogenic PAHs (NAPH, ACNY, CAN, FL, PHEN, ANTH, FLUO, and PYR) are accounted to be at proportion more than 70% of the total PAHs detected in meat samples. It is obvious that the noncarcinogenic PAHs in smoked meat samples are predominant. The genotoxic PAH8 was found to be more than 20% relative percentage of the total PAHs detected and the most predominant PAHs was B[a]P and found to be less than 5% relative percentage.

From Table 5, naphthalene, acenaphthene, fluorine, and phenanthrene (noncarcinogenic PAHs) shows the highest mean concentration of 4.78 ng/g, 25.17 ng/g, 1.97 ng/g, and 1.45 ng/g, respectively, whereas the lowest concentration was found for chrysene, I[cd]P and D[ah]A, comprising less than 5.0% of the total PAH concentration with respect to PAH8. B[a]P (0.90 ng/g; <5.0%) with a frequent detection more than 20%, and I[cd]P was found to be the second highest detected PAHs with more than 15%. From the values reported by EFSA, the highest mean concentration for individual PAH8 was 0.47 ng/g and 0.29 ng/g for CHRY and B[a]P, respectively, in grilled meat samples in EU countries [50]. However, the mean concentration for the sum of PAH8 detected in this method was 1.82 ng/g, which is close to the reported mean concentration (1.77 ng/g) by EFSA [50]. B[a]P was detected in 23% of the total sample analyzed, and the mean concentration was 0.90 ng/g, which is below the maximum allowable limit adopted by the European Union (EU) for B[a]P (5 ng/g) in smoked meat [43, 52]. The maximum limits for the rest of carcinogenic PAHs were not yet established. The mean concentration of PAH8 and ∑B[a]Peq, shows relatively low levels of genotoxic PAHs in smoked meat. The European countries set a maximum level of 2 ng/g for individual PAH and 5 ng/g for the sum of PAH8 in olive residual oil [53].

B[a]P content was found to be with a maximum of 3.63 ng/g, which represents a factor below the level permitted by EU (5 ng/g). However, the mean BaP for all analyzed samples was 0.90 ng/g, which is also far below the permitted limits.

EFSA declared that the PAH4 or PAH8 are significantly more effective indicator of PAHs occurrence in food than the concentration of B[a]P alone [50]. However, in relation with PAH4, the total concentration calculated for different smoked meat was 10.84 ng/g, and for PAH8, the total concentration was 14.54 ng/g (Table 5). B[b]F and B[ghi]P was not found in all tested smoked samples.

### 3.7. Method Uncertainty

Repeatability of the analysis was performed for two levels on different days: ten replicates for each level and the standard deviation were defined. The uncertainty derived from repeatability is calculated by the following equation:

(1) $$\begin{array}{c} U\left(\text{rep}\right)=\frac{\text{RSD}}{V_{n}}, \end{array}$$

where RSD is the relative standard deviation and *n* is the number of repetition (*n*=10). *U*(rec) is calculated and found to be equal 3.32%. *U*(*p*) is the relative standard uncertainty due to precision and expressed as RSDs (%) and found to be 11 %.

Standard uncertainty of the stock standard purity (%) (*U*_STD_) is 0.00577 and the standard uncertainty of the internal standard purity (%) (*U*_INSTD_) is 0.00576.

The combined uncertainty (*U*(*c*)) is calculated by the following equation:

(2) $$\begin{array}{c} U\left(\text{c}\right)=\;\;\mathrm{square}\;\;\mathrm{root}{\left(U\left(\text{p}\right)\right)}^{2}+{\left(U\left(\text{rec}\right)\right)}^{2}+\left(U\left(\text{STD}\right)\right)+\left(U\left(\text{INSTD}\right)\right)=11\text{.}49\%\text{.} \end{array}$$

Expanded uncertainty is calculated by multiplying the combined uncertainty by a coverage factor *k* (*k* = 2 at 95% confidence level) as follows:

(3) $$\begin{array}{c} U_{\exp}=U_{c}\;*\;K,\\ U_{\exp}=22\text{.}98\%\text{.} \end{array}$$

## 4. Conclusions

This study reports for the first time the application of Quenchers for the determination of PAHs in smoked meat samples in Qatar. However, this study reports the levels of BaP, PAH8, ∑LMW, ∑B[a]Peq, ∑PAHs, and ∑HMW in smoked meat items commonly consumed in the Gulf countries. The levels of PAHs in this study found to be not exceeding the maximum levels (according to a regulation of European Commission (EU) no. 835/2011) for PAHs determined in samples [51].

The developed method and performance characteristics are in accordance with the required in legislation. The proposed EU method which defined a range of acceptable recovery between 50 and 120%, with a maximum LODS and LOQ of 0.3 ng/g and 0.9 ng/g, respectively, for B[a]P in food [40, 41].

## Figures and Tables

**Table 1:** Retention time, quantifier ions, and qualifier ions (*m/z*) for target PAHs and d-PAHs.

Number	Analyte	RT (min)	Quantifier ion (*m/z*)	Qualifier ion (*m/z*)
**1**	**Naphthalene-d8**	6.192	136	128/137
2	Naphthalene	6.208	128	102/78
**3**	**Acenaphthylene-d8**	7.746	160	152/161
4	Acenaphthylene	7.746	152	126/76
5	Acenaphthene	7.946	154	152/76
6	Fluorene	8.501	166	115/82
**7**	**Phenanthrene-d10**	9.741	188	158/189
8	Phenanthrene	9.779	178	152/176
9	Anthracene	9.848	178	152/176
**10**	**Fluoranthene_D10**	11.904	212	202/213
11	Fluoranthene	11.950	202	200/101
**12**	**Pyrene_D10**	12.375	212	106/211
13	Pyrene	12.413	202	200/101
14	Benz[a]anthracene	15.349	228	226/114
15	Chrysene	15.453	228	226/114
16	Benzo[b]fluoranthene	18.117	252	126/113
17	Benzo[k]fluoranthene	18.178	252	126/113
**18**	**Benzo[a]pyrene_D12**	18.814	264	132/252
19	Benzo[a]pyrene	18.883	252	126/113
20	Indeno[1,2,3-cd]pyrene	21.409	276	138/274
21	Dibenz[a,h]anthracene	21.486	278	139/276
**22**	**Benzo[g,h,i]perylene_D12**	21.880	288	276/125
223	Benzo[g,h,i]perylene	21.945	276	138/274

**Table 2:** Retention time (Rt), regression coefficient (r2), slope, intercept of the calibration curve, limit of detection (LOD), and limit of quantitation (LOQ).

Compound	RT (min)	R2	Calibration range (ng/g)	Intercept	Slope	LOD (ng/g)	LOQ (ng/g)
Naphthalene	5.208	0.9998	0.5–50	0.3217	0.0877	2.74	6.64
Acenaphthylene	7.746	0.9996	0.5–50	0.4983	0.0221	7.60	20.01
Acenaphthene	7.946	0.9998	0.5–50	0.0193	0.0078	2.10	2.77
Fluorene	8.501	0.9981	0.5–50	0.0335	0.0662	1.44	2.48
Phenanthrene	9.779	1.0000	0.5–50	0.0345	0.1582	1.60	4.21
Anthracene	9.848	0.9998	0.5–50	0.0495	0.1039	0.36	0.87
Fluoranthene	11.950	0.9999	0.5–50	−0.0078	0.115	0.48	1.07
Pyrene	12.413	1.0000	0.5–50	−0.0114	0.0707	0.50	0.83
Benz[a]anthracene	15.349	1.0000	0.5–50	−0.0034	0.112	0.69	1.13
Chrysene	15.453	0.9999	0.5–50	−0.0173	0.1231	0.44	0.62
Benzo[b]fluoranthene	18.117	0.9995	0.5–50	0.0841	0.2466	0.24	0.41
Benzo[k]fluoranthene	18.178	0.9999	0.5–50	0.1379	0.1314	0.53	1.05
Benzo[a]pyrene	18.883	0.9996	0.5–50	−0.0163	0.1667	0.34	0.63
Indeno[1,2,3-cd]pyrene	21.409	1.0000	0.5–50	0.0183	0.1884	0.40	0.75
Dibenz[a,h]anthracene	21.486	0.9998	0.5–50	−0.0461	0.1885	0.63	1.22
Benzo[g,h,i]perylene	21.945	0.9998	0.5–50	0.1116	0.1448	1.71	4.41

LOD = average concentration of the matrix blank + 3 ∗ standard deviation of the matrix blank (*n*=10); LOQ = average concentration of the matrix blank + 10 ∗ standard deviation of the matrix blank (*n*=10).

**Table 3:** : Recovery percentage, relative standard deviation (RSDs, %) and RSDs pooled (%) for the number of determination (*n*=10) for each spiking level.

Compound	Recovery ± RSDs (%)				Pooled RSD (%)
	First day	Second day	First day	Second day	
	1 ng/g	1 ng/g	10 ng/g	10 ng/g	
	Recovery ± RSD%	Recovery ± RSD%	Recovery ± RSD%	Recovery ± RSD%	
Naphthalene	99 ± 6	104 ± 14	101 ± 6	101 ± 6	9
Acenaphthylene	117 ± 24	95 ± 18	99 ± 4	105 ± 7	15
Acenaphthene	111 ± 5	117 ± 16	99 ± 3	103 ± 6	9
Fluorene	101 ± 19	99 ± 16	96 ± 4	106 ± 11	15
Phenanthrene	88 ± 16	107 ± 13	95 ± 5	113 ± 17	14
Anthracene	83 ± 17	106 ± 5	99 ± 2	108 ± 12	11
Fluoranthene	93 ± 11	102 ± 10	98 ± 4	110 ± 15	10
Pyrene	98 ± 7	97 ± 9	95 ± 5	111 ± 16	10
Benz[a]anthracene	100 ± 9	97 ± 9	93 ± 5	110 ± 12	9
Chrysene	101 ± 8	92 ± 11	98 ± 3	110 ± 13	10
Benzo[b]fluoranthene	103 ± 14	96 ± 6	98 ± 3	112 ± 20	13
Benzo[k]fluoranthene	79 ± 38	101 ± 6	100 ± 1	100 ± 11	20
Benzo[a]pyrene	106 ± 10	99 ± 9	98 ± 2	105 ± 18	11
Indeno[1,2,3-cd]pyrene	109 ± 10	74 ± 10	96 ± 2	104 ± 18	12
Dibenz[a,h]anthracene	108 ± 11	92 ± 8	100 ± 1	107 ± 19	12
Benzo[g,h,i]perylene	107 ± 14	97 ± 11	90 ± 4	111 ± 17	12
Recovery range (%)	79–117	74–117	90–101	101–113	
RSDs range (%)	5–38	6–18	1–6	6–20	

**Table 4:** : Levels of individual PAHs detected in different samples (ng/g) (*n*=2).

PAHs	Nap	ACN	FL	PHEN	ANTH	FLUO	PYR	B[a]A	CHR	B[a]P	I_cd P	DB[a,h] A	∑PAHs
Al-Tag tky smoked breast	nd	nd	1.58	nd	1.00	nd	nd	nd	nd	0.39	nd	nd	2.97
Prime tky smkd breast strip	nd	nd	1.73	1.45	nd	0.77	1.05	nd	nd	0.36	nd	0.63	6.00
Prime chk smoked breast	nd	nd	1.47	nd	nd	nd	0.51	nd	nd	nd	nd	nd	1.98
Gourmet smoked chicken roll	2.86	nd	nd	nd	nd	0.77	1.13	nd	nd	nd	nd	nd	4.76
Gourmet smoked turkey	nd	nd	nd	nd	nd	nd	nd	nd	nd	0.35	nd	nd	0.35
5 yildiz smoked turkey breast	nd	nd	1.22	nd	nd	nd	nd	nd	nd	nd	nd	nd	1.22
Volys tky smoked breast	5.73	25.17	2.26	nd	nd	nd	nd	nd	nd	nd	0.75	nd	33.90
Siniora tky smoked breast	nd	nd	nd	nd	nd	0.60	nd	nd	nd	nd	0.87	nd	1.47
Euro gourmet smoked chicken	nd	nd	3.34	nd	nd	nd	nd	nd	nd	nd	0.63	nd	3.97
Volys tky smoked strips	4.70	nd	1.63	nd	nd	0.59	0.84	nd	nd	nd	nd	nd	7.76
Al tag chk m plain	nd	nd	1.91	nd	nd	nd	nd	nd	nd	nd	nd	nd	1.91
Al-Tag tky roast	7.86	nd	3.84	nd	nd	nd	nd	nd	nd	nd	0.41	nd	12.11
Volys chk breast fillet	nd	nd	nd	nd	nd	nd	nd	nd	0.53	nd	nd	nd	0.53
Gourmet smkd ckn breast blk	nd	nd	nd	nd	nd	nd	nd	nd	nd	3.63	nd	nd	3.63
Gourmet ckn plain mortadella	nd	nd	nd	nd	nd	nd	nd	nd	nd	nd	nd	nd	nd
Aia tky oven breast low fat	nd	nd	nd	nd	nd	nd	nd	nd	nd	nd	nd	nd	nd
Al-Tag tky breast	3.49	nd	nd	nd	nd	nd	nd	nd	nd	nd	nd	nd	3.49
Aia chk breast low fat	nd	nd	nd	nd	nd	nd	nd	nd	nd	nd	nd	nd	nd
Euro gourmet rstd chicken fi	3.22	nd	nd	nd	nd	nd	nd	nd	nd	nd	nd	nd	3.22
Aia tky roast thigh	3.65	nd	nd	nd	nd	nd	nd	nd	nd	nd	nd	nd	3.65
Al-Tag beef m smoked	nd	nd	nd	nd	nd	nd	nd	nd	nd	nd	0.42	nd	0.42
Al-Tag beef m roasted smoked	nd	nd	nd	nd	0.36	nd	0.52	1.15	0.94	0.48	nd	nd	3.44
Siniora pastrami w/pe	4.07	nd	nd	nd	nd	nd	nd	nd	nd	nd	nd	nd	4.07
Bibi smoke turkey bre	nd	nd	nd	nd	nd	nd	nd	nd	nd	nd	nd	nd	nd
Sams finest tky bst r		nd	1.38	nd	nd	nd	nd	nd	nd	nd	nd	nd	1.38
Sams finest thy bst g	7.42	nd	1.54	nd	nd	nd	nd	nd	nd	0.37	nd	nd	9.32
Bibi chicken mortadel	nd	nd	1.71	nd	nd	nd	nd	nd		nd	nd	nd	1.71
5 yildiz turkey mortad	nd	nd	nd	nd	nd	nd	nd	0.91	0.58	0.71	nd	nd	2.20
5 yildiz beef mortadel	nd	nd	nd	nd	nd	nd	nd	nd	nd	nd	nd	nd	nd
Gourmet beed pastrami		nd	nd	nd	nd	nd	nd	nd	0.45	nd	nd	nd	0.45
∑PAHs	43.00	25.17	23.61	1.45	1.36	2.73	4.05	2.06	2.49	6.29	3.07	0.63	—
Averg. concentration	4.78	25.17	1.97	1.45	0.68	0.68	0.81	1.03	0.62	0.90	0.61	0.63	—
SD	1.83	—	0.84	—	—	0.1	0.29	0.16	0.22	1.3	0.2	—	—
Frequently detected (%)	30.00	3.33	40.00	3.33	6.67	13.33	16.67	6.67	13.33	23.33	16.67	3.33	—

Note: nd refers to values below LODs.

**Table 5:** Mean concentration of PAHs, ∑PAHs, TEF, TEQ (∑B[a]Peq), and relative % to ∑PAHs (ng/g wet/wt).

Compounds	∑ Indiv. PAHs	min	max	Mean ∑PAHs	TEF	TEQ	Relative % to ∑PAHs	Frequent detection (%)
Naphthalene	43.00	2.86	7.86	4.78	0.000	0.000	32.82	30.00
Acenaphthene	25.17	25.17	25.17	25.17	0.001	0.025	19.22	3.33
Fluorene	23.61	1.22	3.84	1.97	0.001	0.024	18.02	40.00
Phenanthrene	1.45	1.45	1.45	1.45	0.001	0.001	1.11	3.33
Anthracene	1.36	0.36	0.36	0.68	0.010	0.014	1.04	6.67
Fluoranthene	2.73	0.59	0.77	0.68	0.010	0.027	2.08	13.33
Pyrene	4.05	0.51	1.13	0.81	0.001	0.004	3.09	16.67
Benz[a]anthracene	2.06	0.91	1.15	1.03	0.100	0.206	1.57	6.67
Chrysene	2.49	0.45	0.94	0.62	0.010	0.025	1.90	13.33
Benzo[a]pyrene	6.29	0.35	3.63	0.90	1.000	6.289	4.80	23.33
Indeno[1,2,3-cd]pyrene	3.07	0.41	0.87	0.61	0.100	0.307	2.35	16.67
Dibenz[a,h]anthracene	0.63	0.63	0.63	0.63	1.000	0.625	0.48	3.33
∑PAHs	115.92							
∑PAH8^*∗*^	14.54	1.82						
∑PAH4^*∗∗*^	10.84	2.71						
∑LMW^*∗∗∗*^	94.59	15.77						
∑HMW^*∗∗∗∗*^	21.32	2.13						
Mean of the ∑PAHs		3.71						

^*∗*^B[a]A, Chry, B[b]F, B[k]F, B[a]P, Ind[1,2,3-cd]pyrene, Dibenz[a,h]A, and Benzo[ghi]P. ^*∗∗*^B[a]A, Chry, B[b]F and B[a]P. ^*∗∗∗*^Low molecular weight (LMW): Naph, ACNY, ACN, Fl, Phen and Anth. ^*∗∗∗∗*^High molecular weight (HMW): Fluor, Pyr, B[a]A, Chry, B[b]F, B[k]F, B[a]P, Ind[1,2,3-cd]pyrene, Dibenz[a,h]A, and Benzo[ghi]P.
